# What’s counted as a reindeer herder? Gender and the adaptive capacity of Sami reindeer herding communities in Sweden

**DOI:** 10.1007/s13280-016-0834-1

**Published:** 2016-11-22

**Authors:** Astri Buchanan, Maureen G. Reed, Gun Lidestav

**Affiliations:** 1 University of Saskatchewan, 117 Science Place, Saskatoon, SK S7N 5C8 Canada; 2 Department of Forest Resource Management, Swedish University of Agricultural Sciences, 901 83 Umeå, Sweden

**Keywords:** Adaptive capacity, Community resilience, Gender, Indigenous peoples, Reindeer husbandry, Sustainable livelihoods, Sami

## Abstract

Researchers of adaptive capacity and sustainable livelihoods have frequently used social, cultural, human, economic and institutional capitals to better understand how rural and resource-dependent communities address environmental, social and economic stresses. Yet few studies have considered how men and women contribute differently to these capitals to support community resilience overall. Our research sought to understand the differential contributions of Sami men and women to the adaptive capacity of reindeer husbandry and reindeer herding communities in northern Sweden. Our focus revealed a gendered division of labour in reindeer herding as an economic enterprise as well as gendered contributions to a broader conceptualization of reindeer husbandry as a family and community-based practice, and as a livelihood and cultural tradition. Based on our results, we recommend that community resilience be enhanced by generating more opportunities for men to achieve higher levels of human and economic capital (particularly outside of herding activities) and encouraging women to contribute more directly to institutional capital by participating in the formation and implementation of legislation, policies and plans.

## Introduction

“…And what’s counted as a reindeer herder? It’s most of the things that the men do.

And today, I think, reindeer herding is many things. So also it is to learn children and take care of the language – that kind of stuff could be a part of reindeer herding” (Woman, age 18–34).

Addressing the question, “What’s counted as a reindeer herder?”, posed by a Sami reindeer herder in our study, is important for interpreting the contributions men and women make to reindeer herding communities, particularly during times of social, economic and environmental stress. In the past century, climate change, large-scale resource extraction, industrialization, colonization and social and political change have all affected Indigenous peoples living in northern settings. Among the Indigenous people of Sweden—the Sami—is a small proportion that continues to pursue their traditional livelihood by means of reindeer husbandry. While research from Scandinavia suggests that Sami men and women make important, yet differentiated, contributions to their community (Kuokkanen 2009; Andersson and Keskitalo 2012), few researchers have connected these observations to examining the gendered dimensions of the adaptive capacity of Sami communities. Our research reveals that narrow assumptions about what activities constitute reindeer herding and who undertakes them typically focus solely on men as herders. These assumptions erase from view multiple contributions of both women and men who seek to sustain the business as well as the community and cultural practices associated with reindeer husbandry. As a consequence, we may not fully understand the assets or capitals used to build adaptive capacity of reindeer herding communities or what members of those communities are best positioned to contribute to community resilience.

The purpose of this paper is to understand the contributions of Sami men and women to the adaptive capacity of reindeer husbandry and the resilience of reindeer herding communities in northern Sweden. Specifically, we address the following questions: (1) How might reindeer husbandry be conceptualized to account for the multiple contributions of women and men to the capacity of Sami communities to adapt to environmental, social and economic change? (2) What can a focus on the assets or capitals of reindeer herders reveal about how women and men contribute to their adaptive capacity and livelihoods at individual and household levels and the resilience of Swedish reindeer herding communities?

## Background

### Key stresses facing Scandinavian reindeer herders

The 20th century brought rapid environmental, economic, social and political changes to the Sami of northern Sweden (Berg 2010; Löf 2014; Össbo 2014). Nuclear weapons testing between 1940 and 1960s reduced the demand and price of reindeer meat (Bostedt 2001). The effects of the Chernobyl nuclear disaster of 1986 fell heavily upon residents of northern Sweden and reinforced a public concern over the presence of radiocaesium in both food sources for reindeer as well as in the resulting meat products (Åhman et al. 1990; Bostedt 2001). More recently, variations in weather and seasons, due to climate change, have resulted in changes in movement patterns of the herds, impacted vegetation for fodder and altered breeding cycles (Furberg et al. 2011). Although climate change has sparked considerable research on adaptive capacity, studies of the reindeer herding communities of Norway and Sweden indicate that climate change impacts may be less detrimental to the overall adaptive capacity of Sami people than the complex economic and socio-political environment within which they must manoeuver (e.g. Tyler et al. 2007; Löf 2014). In Sweden and Finland, resource use conflicts between reindeer herding and the forestry industry may be more significant (Keskitalo 2008; Raitio 2008; Berg 2010).

Despite protections for reindeer herding in Swedish law, industrialization and resulting land-use conflicts have also restricted reindeer herding enterprises and communities. The cumulative effects of forestry, wind and hydropower, mining, and infrastructure development has, among other management problems, resulted in an extensive reduction of the availability of winter grazing land (lichen abundant forest) and access to migratory paths. Further, the dialogue between the reindeer herding communities, other land users and state agencies has been inadequate. To overcome this problem, a stakeholder-driven process was initiated in 1999 to develop land-use plans for reindeer herding, named Reindeer Herding Plans (RHPs) (Sandström 2015). Fifteen years later, 50 of Sweden’s 51 reindeer herding districts have undertaken planning, using RHPs to plan their daily activities, as a tool for discussions with various types of land users, and to communicate and show reindeer herding activities (Vestman 2014).

Finally, social challenges facing Sami reindeer herders include assimilation policies by the dominant societies that began in the 19th century. Policies introduced in the name of social welfare and education undermined basic rights and altered Sami way of life (Kvist 1994; Kuokkanen 2009). The Sami population dwindled, traditional familial roles blurred and the number of people who spoke the Sami language declined. While assimilatory policies have long been abandoned, Sami people believe that their lifestyle and ethnic identity remain at risk (Amft 2000; Omma et al. 2012).

Amidst these changes, gender roles and relations among the Sami have also changed and their interpretation varies. For example, Kuokkanen (2009) argued that prior to Scandinavian government intervention, men and women were treated with equal privilege and had equal rights with regard to property ownership and inheritance in reindeer herding communities. Since the 1960s, the push to make reindeer herding more economically profitable has led to rationalization, mechanization and an increased masculinization of the enterprise. According to Amft (2000), this push has also resulted in emphasizing the practical aspects of reindeer herding as part of the male domain and reducing the involvement of women. Amft even argues that women have had to subordinate themselves as women in order to be viewed as “genuine” Sami, thereby “contributing to their own marginalization and helping to maintain their subordinate position in Sami society” (Amft 2000, p. 202). Researchers have found that Sami women’s access to property has been restricted (Li and Singlemann 1998; Kuokkanen 2009; Amft 2000) and, over time, many women have left the ‘traditional Sami lifestyle’ in search of gainful employment or education elsewhere (Kuokkanen 2009).

### Conceptualizing adaptive capacity and resilience among Sami reindeer herding communities

In this study, we define communities operationally as Sami communities involved in reindeer husbandry. As described below, reindeer husbandry is a livelihood practice that includes cultural traditions and experiential knowledge. Different forms of capital contribute to adaptive capacity at the individual and household level. These contributions, in turn, support sustainable livelihoods of households. The mobilization of adaptive capacity to support community resilience requires processes that scale up from the household to the community level. Factors and processes that can contribute to mobilization include, but are not limited to, gender relations, legislation and policies, capacity building, social learning and leadership. Mobilization processes may be limited by uneven power relations and institutional norms and structures associated with those factors. Our focus in this paper is primarily on gender relations, although we recognize that gender interacts with other factors and processes.

#### A framework for reindeer husbandry

**Fig. 1:**
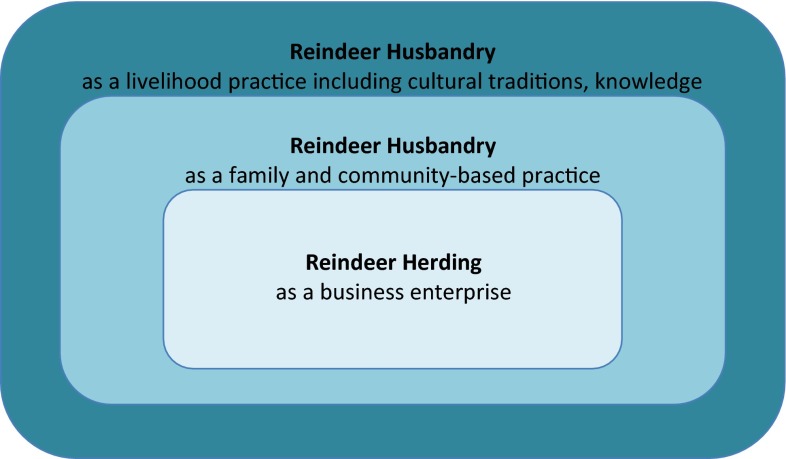
Functional scales of reindeer husbandry in Sweden

In 2009, the Sami Parliament reported that 85 % of reindeer herders were men and 80 % of reindeer were owned by men (Sami Parliament 2009). It is unclear what activities were considered within the herding enterprise when these data were presented. Herders themselves consider reindeer husbandry a way of life, with associated knowledge and cultural traditions (Svenska Samers Riksförbund [National Association of Swedish Sami] 2015). Hence, we conceive of reindeer herding within the broader realm reindeer *husbandry*—a concept that involves three functional levels—the business enterprise of herding, the land-based practices of families and communities, and the practices and cultural traditions that characterize reindeer husbandry as a livelihood (Fig. 1). Broadening the conceptualization of reindeer husbandry to consider practices rooted in family, community, livelihood and culture acknowledges the broader significance of husbandry practices and allows the opportunity to consider different ways women and men contribute to the adaptive capacity of their households and communities.

#### Situating adaptive capacity within an understanding of community resilience

Adaptive capacity has been conceptualized by Scandinavian researchers as a component of the broader concept of community resilience (e.g. Keskitalo 2008; Löf et al. 2012)—a concept with multiple definitions (Community and Regional Resilience Institute 2013). We have adopted the following definition of community resilience: “existence, development and engagement of community resources by community members to thrive in an environment characterized by change, uncertainty, unpredictability and surprise” (Magis 2010, p. 401). This definition shares common attributes of the sustainable livelihoods framework that has been developed to identify a “critical mass of assets needed to cope with stresses and shocks and to maintain and enhance capabilities now and in the future” (Tamarack 2016, p. 1). Both definitions imply the need to determine and characterize a set of assets, processes and feedbacks that can be harnessed by community members during uncertain times. Importantly, understanding community resilience requires understanding how assets are accessed and used at multiple levels. Researchers have found that households that are able to sustain their livelihood will likely contribute to community and regional resilience, but the mechanisms by which they do so are not specified (Marschke and Berkes 2006; Ross and Berkes 2013; Kiewisch 2015; Smyth and Sweetman 2015).

Feminist scholars of resilience, adaptation and development point out that gender dynamics are central to the ways household members earn, control and allocate resources; hence, understanding gender roles and relations can help us understand the role of women and men in production or employment, their access to specific assets and the ability to influence access to and/or management of resources and assets beyond the household (Smyth and Sweetman 2015). Men and women contribute to their household’s livelihood in different ways and have different gendered obligations to meet both within their household and within their larger communities. Opportunities and vulnerabilities for men and women alike are shaped by life-stage and structural factors, including taken-for-granted gendered patterns and practices. An understanding of differential contributions by different members of the household, therefore, can help us better understand their adaptive capacity and community resilience as a whole.

We define adaptive capacity as the ability of a system to continually develop and alter itself in the face of change without sacrificing its most important functions (after Folke 2006; Smit and Wandel 2006). We examine capacity to adapt with reference to assets available to be mobilized after other studies assessing adaptive capacity and sustainable livelihoods (e.g. Wall and Marzall 2006; Cutter et al. 2008; Klenk et al. 2012). Initially, the sustainable livelihoods framework focused on five assets: financial, social, human, physical and personal (e.g. Department for International Development 1999); however, variations among capitals selected for study also exist (e.g. Scoones 2009). We followed the advice that cultural capital is important (Adger et al. 2013), particularly for Indigenous communities that continue to rely on natural resources as a foundation for their livelihoods.

Here, we report on five forms of capital: social, institutional, human, economic and cultural. *Social capital* refers to networks, norms and trust relations that allow people to work effectively together to pursue shared goals (Coleman 1988; Portes 1998). Bonding forms of social capital help community members connect internally, while bridging forms of social capital help connect communities with outsiders. *Institutional* capital is defined as institutions and governance structures (Platje 2008). *Human* capital refers to knowledge and skills of individuals that can be used for economic or political advantage, typically indicated by educational attainment (Becker 1993). *Economic* capital is typically expressed in terms of monetary income and assets (Anheier et al. 1995). Finally, *cultural* capital can take the form of embodied traits or skills, material assets or education (Bourdieu 1979). Indicators and variables defining these capitals were developed based on relevant literature described above and in-depth discussions with Swedish researchers who had lived and/or worked in Sami communities.

## Materials and methods

**Fig. 2:**
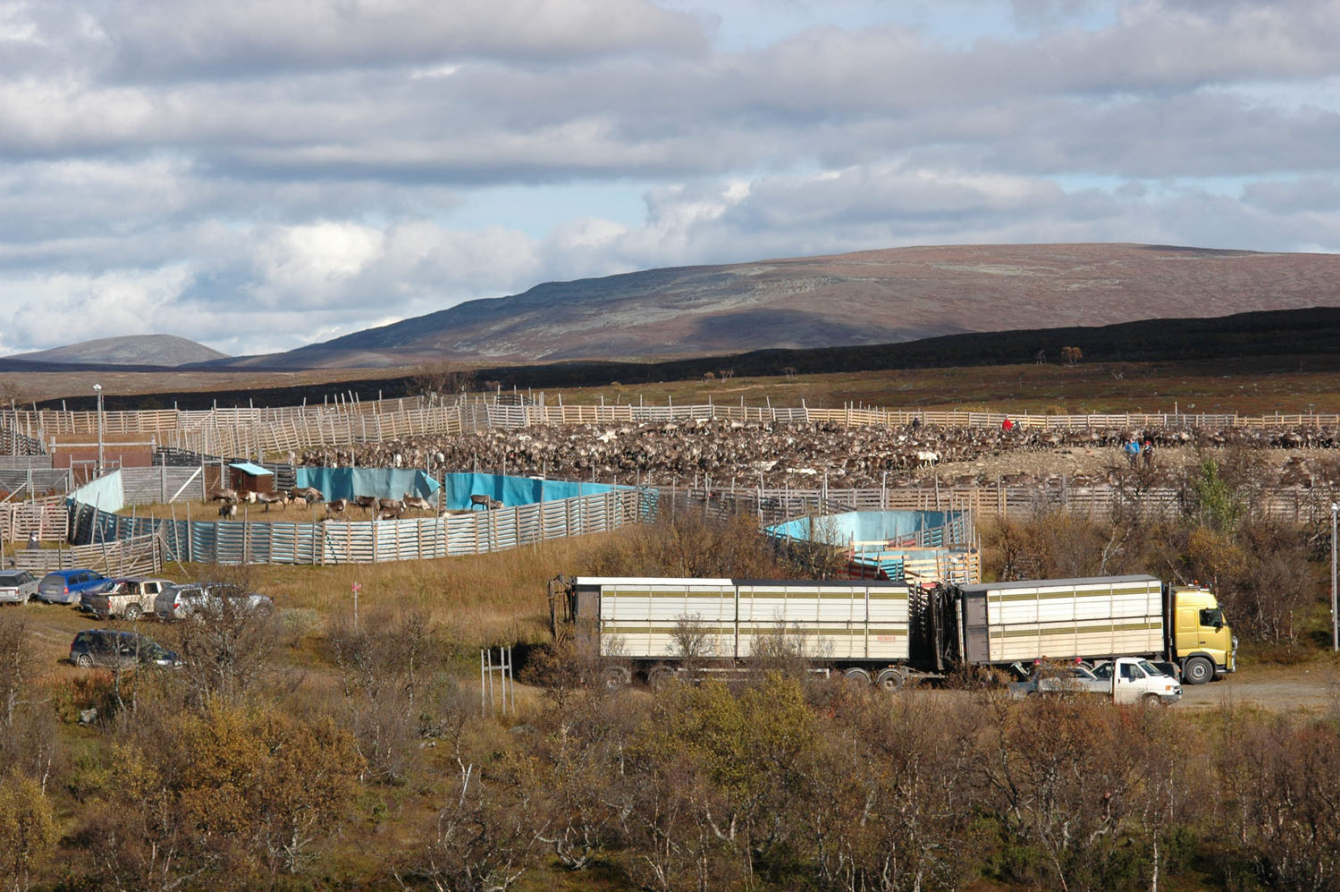
Reindeer herding corral in the study area (Photographer: Maureen Reed)

Adato and Meinzen-Dick (2002) suggest a mix of quantitative and qualitative data is necessary to understand livelihoods, specifically pointing to the value of combining results from household surveys, interviews, focus groups and secondary sources. All of these sources were used in this study. Quantitative and qualitative data were collected by the first author using a questionnaire survey of reindeer herding communities in Sweden involved in herding plans. There are 51 reindeer herding communities in Sweden; all were invited to participate in the study and 34 agreed (Fig. 2). Questionnaires (in Swedish) were mailed to 270 households. Eighty-one questionnaires were returned from 63 households, for a household response rate of 23.3 %. Of respondents, 59 were men and 22 were women.

Numerical data from the questionnaires were analysed using SPSS (V. 22). Frequencies were the most important statistical measures, as expressed by the percentage of responses to each question. Male and female respondents’ answers were compared to each other using Chi-square tests. A *P* value of 0.05 was used as a cutoff between significant and non-significant results.

Nine respondents to the questionnaires agreed to follow-up interviews conducted by the first author with assistance of a Swedish student who was undertaking her Master of Science thesis work in parallel with this study. Interviewees were members of one of the reindeer herding communities in Vilhelmina (Vilhelmina Norra or Vilhelmina Södra). The region has a population of about 7500 people and is subject to a variety of land uses, including forestry and Sami reindeer husbandry as well as a growing number of hydroelectric projects, and mining and tourism operations (Sandström 2015). The subsequent interviews were done in four parts: one focus group with five individuals (two men and three women), one double interview (two men), and two single interviews, each with women. Initially, the interviews were to be conducted individually; however, due to scheduling difficulties, this was not possible. Interviews were conducted in the municipality centre of Vilhelmina, at the Model Forest Office, in English and Swedish. Due to the small number of interviews, these responses were manually analysed for key themes and were used primarily to explain trends found through the questionnaire responses. Secondary data sources were also analysed including income data from the Vilhelmina Tax Office, as well as brochures, posters and articles made available by the Vilhelmina Model Forest staff (VMF).

## Results

### Assessing gendered contributions to reindeer husbandry

**Fig. 3:**
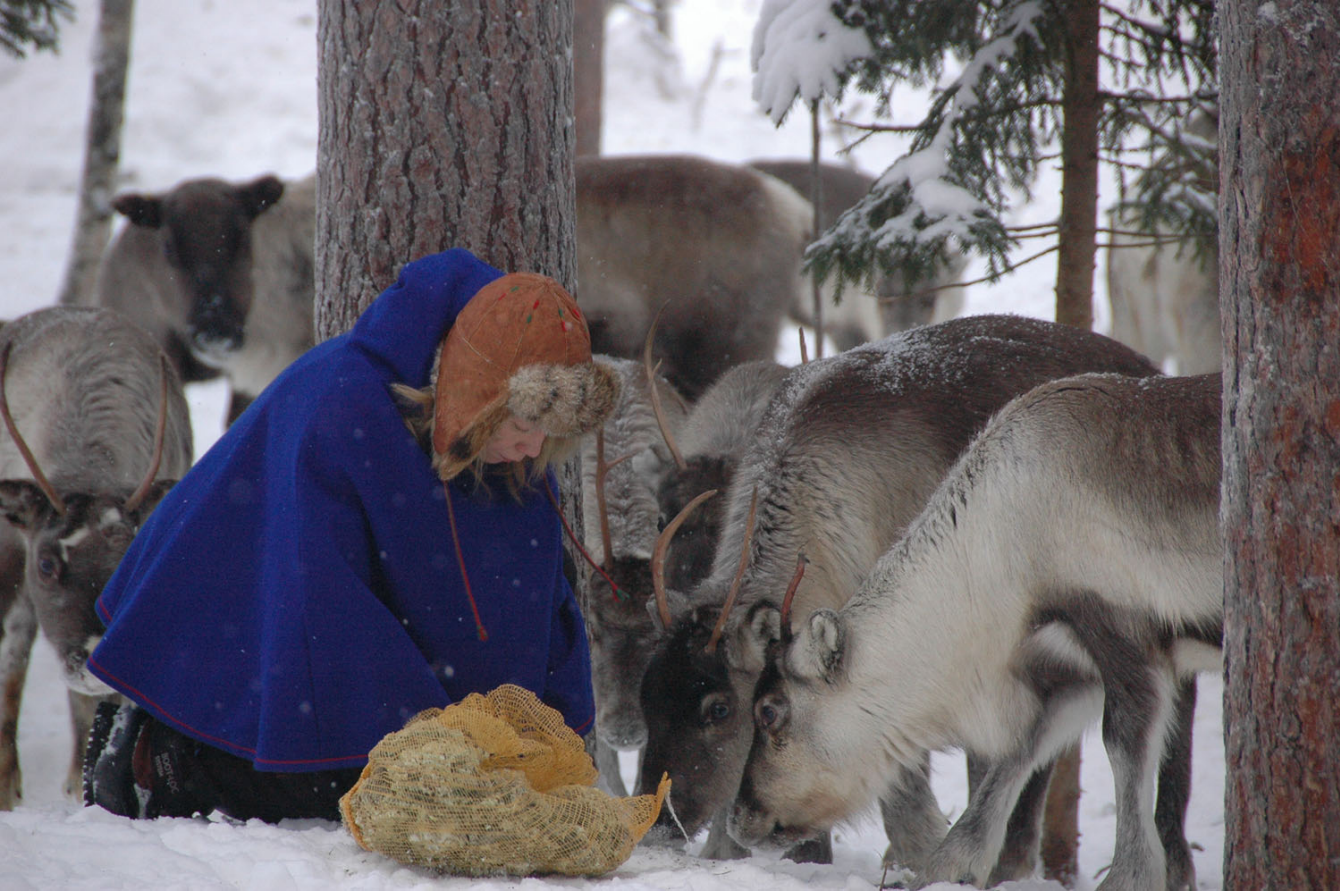
Reindeer herding woman providing supplemental food (ground lichens) during reindeer migration (Photographer: Ursula Neussel; Swedish Forest Agency)

Rural communities that rely on natural resources around the world demonstrate a highly gendered division of labour (e.g. Reed 2003; Neis et al. 2005; Alston 2011) and pastoralists like reindeer herding communities appear to be no exception (Fig. 3). Young and older female and male interviewees reported that there were different expectations for men and women in households regarding who should and how to support reindeer husbandry:

“Today, it doesn’t matter—the parents give both daughters and sons the same possibilities. So they, of course—but often it’s a hard work, it’s easier for boys to take part of the work. You see I have a son, he’s 12 years old, he is sitting on the motorbike now for, I think, his third year. So it comes to [be] a man’s work but they (women and girls) can [come] with us when we are working with the reindeer. But then her real hobbies is horses and dolls and that…” (Man, age 45–54).

“…and I think it’s safer when we’re around […] But it’s sad because—reindeer herding—it’s kind of a macho, a man work today, at least, like, the daily work with it, and it’s sad because I think we lost much knowledge and that kind, from not females in the work every day in winter” (Woman, age 18–34).

The questionnaires provided evidence that social, human, economic and cultural capitals were important elements of the adaptive capacity of Sami herding communities and exhibited gender differences. As one way to indicate gendered dimensions of social capital, we explored long-term attachment to community by asking survey respondents who they anticipated would carry on their reindeer herding business after they retired. Of the 20 (24.7 %) respondents that had both sons and daughters, 45 % stated that both their sons and daughters would inherit their business, 30 % said only their son(s) would inherit and 5 % said only their daughter(s). Focus group interviewees suggested that women have a greater tendency to move away from the community. This observation was supported by their higher levels of formal education as described below.

These differences, however, do not necessarily reflect inequality. Indeed, no survey respondents or interviewees reported that their roles in reindeer herding were unequal. By contrast, men and women shared common perceptions that reindeer herding was misunderstood and disregarded by the dominant Swedish society. The following exchange among three participants of the focus group demonstrates a shared perception of their status within broader society:

“I feel like it’s, they have no respect for our life. They just say, well, we want to put up uh, windmills. [vindkraft]. And we say no, you can’t do that, because then we have no food for the reindeer. They will give us money, but money can’t fix it. Because the money doesn’t do that we have more land. It’s—it’s nothing. And they’re like, yeah well it does” (Man, age 18–34).

“I think they actually don’t know much about our lifestyle or how we work with the reindeers and how the Sami people have lived, how our culture is. I think this is the biggest problem. They don’t know…” (Woman, age 18–34).

“But some of the Swedish people they, they, they think that we live in […] teepees! They don’t know that we live indoors. They think that we do things like… they don’t know that we can drive a car or watch TV in a house, they think that we’re all living in the forest in a teepee. That’s their life. They don’t know that we actually know the outside world” (Man, age 18–34).

“I think really this society see that the Sami culture as a threat…” (Woman, age 35–44).

Interviewer: “A threat?”

“Yeah, about the resources. Natural resources—because they want to keep track of us. … And I think it’s a power thing, because when you compare us to wind power, waterpower, we don’t give the society money in the same way. And that’s why they prefer to have wind power and waterpower in the Sami country. They will always put… be sure that they can handle it in some way” (Woman, age 35–44).

These findings suggest relatively high levels of bonding forms of social capital and lower levels of bridging forms of social capital. Sami reindeer herders continue to see themselves as distinct, yet marginal, actors within the institutional system that regulates their livelihoods. This sense of alienation helps explain the lower levels of institutional capital observed, as measured by the involvement of Sami herders in political life. Voting patterns and participation in official reindeer herding planning processes were both gauged. Importantly, the survey revealed that 95.9 % of 74 respondents cited some form of participation in reindeer herding planning process (100 % of men and 85.0 % of women (*χ* ^2^ = 8,44; *P* = 0.004)). This is important for how Sami people may be able to gain sufficient influence to maintain their way of life in the face of competing land uses and users in the region. Notwithstanding a high level of participation, individuals were not convinced of their influence. One interviewee stated the challenge in this way:

“The Forestry Act doesn’t benefit us; it’s more for the land/forestry owners. It says in it that there should be consultations but they have been very few. Sure there have been some but when an area have been saved the next years they can use it anyway” (Woman, aged 65+).

With respect to human capital, we examined both the transfer of skills between generations and formal levels of education. The survey results revealed that 40.7 % of the sample participated in training, indicating a strong transfer rate of traditional knowledge within reindeer herding communities. There were no differences reported by gender. However, this changed when we focused on more formal education (considered as human capital). Women in the sample were much more likely than men to obtain post-secondary education –72.7 % compared to 11.9 % (*χ* ^2^ = 29.2; *P* < 0.001). Men in the sample were also twice as likely to perceive barriers to higher education (men = 27.1 %, women = 13.6 %) although this difference was not statistically significant (*χ* ^2^ = 1.62). Interviewees revealed that gendered expectations weigh heavily on boys. For example:

“I think it’s a little bit like boys, they feel a lot of pressure, and they stop studying with high school. They have to go work. But girls they don’t have the same pressure, so they can take time to study more” (Man, age 18–34).

Skills and education also connected to economic capital. A statistically significant difference existed between men and women with respect to the number of jobs they held. Forty-two percent of the men (*n* = 25) reported having four or more sources of income compared to only 4.5 % of women. Tax information for the 2011 fiscal year revealed women in the sample tended to make significantly more money than men. The average income for 42 men was USD 13 491, with a range between 0 and 37 507, while 26 women had an average income of USD 22 384, with a range between 4411 and 61 824. Overall, individuals in the sample derived 52 % of their personal income from reindeer herding. Of this total, men derived an average of 69 % of their personal income from entrepreneurial activities, compared to only 25 % for women.

Access to cash within the household contributed significantly to the well-being of children and quality of life for the household. One interviewee explained:

“The wife is working with something else. So she’s the one who pays for the children and that kind of stuff. And they want to go on holiday, have 2 cars, that kind [of thing]—then the wife’s supposed to work for that” (Woman, age 18–34).

To understand the activities that contributed to cultural capital, the questionnaire asked respondents to record their individual participation or the participation of family members for 19 specific activities involved in reindeer husbandry. Our inquiries revealed a broad range of contributions, many of which were gender specific (Table 1).

**Table 1:** Participation in reindeer husbandry activities by gender. *P* values in bold type are considered statistically significant (*P* < 0.05)

Activity	No. of participating respondents	Percentage of men participating	Percentage of women participating	χ2	*P* value
Calf marking	80	100	95.2	2.67	0.092
Business administration	73	84.9	60.0	5.26	**0.022**
Gathering herd	79	93.2	15.0	80.8	**<0.001**
Separating the herd	80	98.3	90.5	2.40	0.105
Moving the herd	77	98.2	76.2	12.05	**0.005**
Fencing work	75	94.6	36.8	32.07	**<0.001**
Feeding reindeer	71	94.2	84.2	2.13	0.179
Coordinate activities for reindeer herding community	71	96.1	60.0	16.83	**<0.001**
Consulting with other land users	69	87.8	50.0	10.33	**0.001**
Teaching and training others	47	71.4	66.7	0.12	0.756
Slaughtering reindeer	77	96.5	90.0	0.41	0.260
Preparation of meat for the household	71	90.2	70.0	4.42	**0.034**
Preparation of meat for sale	44	87.9	63.6	2.94	0.071
Hunting	76	100.0	60.0	18.75	**<0.001**
Fishing	72	94.4	94.4	0.00	1.000
Fuelwood preparation	75	100.0	70.0	15.53	**<0.001**
Gathering berries, mushrooms and wild plants	67	74.5	100.0	5.48	**0.013**
Making of handicrafts for household use	43	77.8	87.5	0.02	0.428
Making of handicrafts for sale	28	52.9	90.9	4.41	**0.036**

Overall, household participation in these tasks was high; 80 % of households contained at least one individual participating in each activity. This indicates a continuation of reindeer husbandry as an entrepreneurial, family and community-based endeavour where traditional knowledge is transferred inter-generationally. A gendered picture also emerged; men reported contributing the most to the physically demanding fieldwork required in reindeer herding enterprises. They participated most in tasks such as gathering the herds, fencing work and hunting, and also played a central role in activities related to the business of herding, including business administration and consultations with other land users. Few women reported participating in gathering herds and fencing work, but they did participate in key activities such as calf marking, feeding and separating the herds. Many more reported being involved in harvesting plants, mushrooms and berries to contribute to family food supply and in creating handicrafts for sale, supplementing the family income. This division of labour was reinforced by interviewees as explained by this man:

“You can say, the men are working in the mountains. With the machines and doing the hard work. But also the women, they take care of the family, the economy, […] to fix the logistic in the Sami village. We need food for the reindeer—someone has to drive it from one place to another—the women… if we take one day when we’re moving a reindeer herd from one corral to another corral, for example when they moving from the forest up to the mountains, and we put out 10,000 kilos of food, often it’s the women’s work to carry that. From one place to the car, from the car to the other place, and out to the reindeer. So, they also have hard work. But they take care about the logistics” (Man, age 45–54).

## Discussion

Our results support others’ findings that social and cultural capitals are key factors for sustaining livelihoods of households and enhancing community resilience (e.g. Adato and Meinzen-Dick 2002; Emery and Flora 2006; Scoones 2009; Adger et al. 2013). A community-level assessment reveals that reindeer herding communities exhibit strong levels of cultural capital that help support human and social capital. This reinforces the importance of paying attention to the cultural assets when assessing livelihoods and resilience in the face of change (Adato and Meinzen-Dick 2002; Adger et al. 2013). Although reindeer herding does not generate high individual or household incomes, pluri-activity of Sami householders contributes to their economic capital and offers an adaptive strategy to the environmental and social changes they are experiencing.

Our research reveals that men spend the greatest amount of time overall with the reindeer in the field, contributing landscape knowledge, physical strength and technical skills for maintaining equipment and in overall business administration. As such, their greatest contributions lie within the innermost functional level of reindeer herding as a business enterprise. Women, on the other hand, contribute more modestly to this level, but are most likely to do so during the busiest times of year. However, their contributions through household earnings enable the business practice to continue. They also participate in the larger functional levels. Women spend the most time child rearing, have stronger proficiency in Sami languages and contribute most strongly to the creation and sale of handicrafts. In these ways, women contribute significantly to reindeer husbandry as a family and community-based practice and as a livelihood and cultural tradition. Hence, our broader conceptualization of reindeer husbandry (Fig. 1) makes visible the multiple contributions of both women and men to the assets necessary to sustain livelihoods and contribute to community resilience (Magis 2010).

The gendered division of labour among Sami householders supports present-day adaptation to the realities of reindeer husbandry. Pressures such as climatic variations, habitat fragmentation, price fluctuations and restrictive legislation have made it more difficult for families to survive financially on reindeer herding alone. Hence, reindeer herding families have adapted within their cultural norms to provide women with the opportunity to seek education and obtain high monetary recompense for external employment, while men engage in the physical work of herding. Both are involved in culturally fulfilling practices of reindeer husbandry in order to sustain their livelihoods and benefit the household overall. While this division of labour is gendered—as is the case farms operated by Swedish families (see Andersson 2014)—it appears, at present, to be functional. Additionally, interviewees pointed out that this phenomenon does not solely take place within a ‘traditional’ family unit, where a mother rears children and works while a father tends to the family’s reindeer herd. Interviewees explained that such an arrangement may also take place in cases where a young woman’s father or brother tends to the reindeer she owns while she is away at school or for other reasons. Yet, neither women nor men who participated in the study identified that their roles in reindeer herding were unequal. This might be interpreted as support for the claims put forward by Amft (2000) that gender equality between Sami women and men is a myth and Sami women must subordinate themselves as women in order to be considered “genuine” Sami. Further research might need to be conducted to determine if this perspective is widely shared among Sami or other researchers.

Gender expectations lead men to carry on the family enterprise, but also restrict their choices regarding education and their resulting employment opportunities. The long-term capacity to adapt, however, may be jeopardized by the present arrangement, as exemplified by the larger gender difference in post-secondary education and the continued efforts of men to seek employment in other resource-based industries. These choices may make men more vulnerable to fluctuating economic and climatic conditions that directly impact these industries and less qualified for other forms of employment. In the event that their reindeer herding enterprise fails, then, men may well be disproportionately disadvantaged as they lack the training to easily find employment elsewhere or may be locked in due to gendered expectations and identities as “primary producers”. This disadvantage for men has been reported in other resource sectors—by Alston (2011) who documented the stresses faced by rural Australian men and women facing challenges in maintaining agriculture during prolonged droughts, by Power (2005) who reported on the restructuring of social relations following declines in Atlantic fisheries and by Reed (2003) who documented the gendered effects of land-use change for those relying on forestry on Canada’s west coast. In all cases, men experienced greater losses of human and economic capitals than women when access to the primary resource declined. As Sami women in this study reported higher average incomes, higher levels of formal education and higher overall language proficiency than Sami men, women are also more likely to have a higher level of personal resilience. This finding illustrates the value of a multi-level analysis as a focus on the household or community level alone may have masked these individual differences.

Differences between men and women regarding the division of labour and inheritance may help explain the observed differences between genders in relation to institutional capital. Although gender differences in institutional capital were not as pronounced as for other forms of capital, men were more involved with RHPs than women. Part of the explanation for this is that the masculine norm of reindeer herding gives men the primary responsibility for making planning decisions. Further, RHPs focus on lands allocated for grazing. Land allocation is more central to the inner core of reindeer husbandry—the business enterprise—rather than the larger functional levels such as family practice and cultural traditions. However, it should also be acknowledged that the RHP is a tool to communicate and generate dialogue about the overall conditions for reindeer husbandry, both within the reindeer herding community and with other land users who may impact conditions for reindeer husbandry (Vestman 2014; Sandström 2015). Given the fact that women have greater formal education and greater language proficiency, they are well positioned to contribute more to institutional capital, either with developing or implementing RHPs, or by participating in other governance institutions that support reindeer husbandry. Their higher levels of human capital, therefore, may translate to more power within and/or outside their communities in the future. This contribution may be critical for long-term community resilience, as it seems impossible for herding communities to become sustainable unless they are able to use institutional capital more effectively to change policies and plans to support the habitat needs for reindeer and the cultural traditions associated with husbandry.

Our research confirms other feminist-inspired scholarship that suggests that understanding household dynamics is necessary for understanding community resilience (Neis et al. 2005; Alston 2011; Smyth and Sweetman 2015). Without drilling down to uncover the dynamics *within* the household, identifying household “averages” may mask important differences between women and men. By focusing on how contributions to adaptive capacity are gendered within households, we have drawn attention to power dynamics at multiple levels—including within households and between communities and government regulators. But the pathways for scaling up from households to communities remain unspecified. Better understanding of processes of mobilization and feedbacks associated with all of the capitals requires attention to a range of processes such as capacity building or enhancement (Emery and Flora 2006), policies and legislation (Adato and Meinzen-Dick 2002) and social learning (Egunyu and Reed 2015). We have touched on some, but not all, of these issues. Better understanding of these processes will require attention to how power circulates within communities and between communities and higher orders of governance (see Ross and Berkes 2013). Research about how processes and pathways are shaped by and contribute to gendered norms and relations is one possible means by which to understand power dynamics and offers a fruitful avenue for future research.

## Conclusions and recommendations

While reindeer herding appears to be a male-dominated industry on the surface, both women and men are active in reindeer husbandry, and their respective roles are invaluable to its success. The research reveals key differences, however, that can be addressed at a policy level to enhance adaptive capacity and livelihoods in support of community resilience. The results indicate that policies that would encourage men to consider appropriate, higher education options would increase their human capital directly, develop the value chain that the reindeer husbandry represents and thereby improve the economic capital derived from a very exclusive business. Such education opportunities must be developed in consultation with men who might benefit from the programmes. Increased education and employment options might offer better opportunities for households and communities to build capacity for resilience, although they may also run the risk of diverting more people out of husbandry altogether, thereby weakening the sustainability of the business of herding and the culture of husbandry and reducing community resilience.

Further, encouraging women to engage in planning processes associated with RHPs would increase institutional capital among reindeer herding communities. The increased level of formal education among women in the sample suggests that women may be well positioned to become more influential in institutions that affect reindeer husbandry, including RHPs, local government and Sami Parliament. This would serve to shift power dynamics in reindeer herding communities, make women’s roles more visible and improve access by reindeer herding communities to a greater pool of intellectual potential to address their relative weakness within the power relations that shape the regulatory environment in which they operate.

Until now, the narrow conceptualization of reindeer husbandry solely as a rural economic enterprise has omitted many of the potential contributions that women and men might make to sustaining their livelihoods and the resilience of their communities. By pursuing a methodology that segregated contributions by gender, this study revealed a more nuanced understanding of the contributions and risks to adaptive capacity, demonstrating how gendered norms and expectations impact the life choices of both men and women, and pointing to the need for gender-sensitive policies and programmes in education, and in processes associated with creating and implementing RHPs. Understanding the present-day gendered contributions of adaptive capacity for sustaining livelihoods and building community resilience provides insights that can help Sami people and researchers to understand and build more sustainable and resilient communities in the future. As gender intersects with a wide range of other mobilizing factors and processes imbued with power relations, this study represents only a first step towards gaining this understanding and taking action.
